# Genetic association between APOE*4 and neuropsychiatric symptoms in patients with probable Alzheimer's disease is dependent on the psychosis phenotype

**DOI:** 10.1186/1744-9081-8-62

**Published:** 2012-12-27

**Authors:** Drew Christie, Jane Shofer, Steven P Millard, Ellen Li, Mary Ann DeMichele-Sweet, Elise A Weamer, M Ilyas Kamboh, Oscar L Lopez, Robert A Sweet, Debby Tsuang

**Affiliations:** 1 VISN-20 Mental Illness, Seattle, WA, USA; 2 Geriatric Research, Education and Clinical Centers, Seattle, WA, USA; 3 Department of Psychiatry, University of Pittsburgh, Pittsburgh, PA; 4 Department of Neurology, University of Pittsburgh, Pittsburgh, PA, USA; 5 Department of Human Genetics, University of Pittsburgh, Pittsburgh, PA, USA; 6 VISN-4 Mental Illness, Research, Education, and Clinical Center, VA Pittsburgh Healthcare System, Pittsburgh, PA, USA; 7 Department of Psychiatry and Behavioral Sciences, University of Washington, Seattle, WA, USA

**Keywords:** Alzheimer’s disease, Apolipoprotein E, Genetics, Behavior, Hallucinations

## Abstract

**Background:**

Neuropsychiatric symptoms such as psychosis are prevalent in patients with probable Alzheimer’s disease (AD) and are associated with increased morbidity and mortality. Because these disabling symptoms are generally not well tolerated by caregivers, patients with these symptoms tend to be institutionalized earlier than patients without them. The identification of protective and risk factors for neuropsychiatric symptoms in AD would facilitate the development of more specific treatments for these symptoms and thereby decrease morbidity and mortality in AD. The E4 allele of the apolipoprotein E (APOE) gene is a well-documented risk factor for the development of AD. However, genetic association studies of the APOE 4 allele and BPS in AD have produced conflicting findings.

**Methods:**

This study investigates the association between *APOE* and neuropsychiatric symptoms in a large sample of clinically well-characterized subjects with probable AD (n=790) who were systematically evaluated using the Consortium to Establish a Registry for Alzheimer’s Disease (CERAD) Behavioral Rating Scale for Dementia (BRSD).

**Results:**

Our study found that hallucinations were significantly more likely to occur in subjects with no *APOΕ*4 alleles than in subjects with two Ε4 alleles (15% of subjects and 5% of subjects, respectively; p=.0066), whereas there was no association between the occurrence of delusions, aberrant motor behavior, or agitation and the number of Ε4 alleles. However, 94% of the subjects with hallucinations also had delusions (D+H).

**Conclusion:**

These findings suggest that in AD the Ε4 allele is differentially associated with D+H but not delusions alone. This is consistent with the hypothesis that distinct psychotic subphenotypes may be associated with the *APOE* allele.

## Introduction

Alzheimer’s disease (AD) is a neurodegenerative disorder that is characterized by a decline in cognitive function, most notably in the areas of short-term memory and learning. Between 30% and 60% of patients who are affected with AD also develop psychotic symptoms (i.e., hallucinations or delusions) during the course of their illness [1]. Psychotic symptoms in these patients are associated with aggression, which results in earlier and more frequent institutionalization compared to patients who are free of psychotic symptoms [2,3]. Therefore, it is critical to understand the genetic, environmental, and medical factors that may increase the risk for developing psychosis in patients with AD.

One well-known risk factor for AD, particularly for late-onset AD (LOAD), that may increase the risk of developing psychosis in patients with AD is the E4 allele of the apolipoprotein E (*APOE*) gene [1]. At least 26 studies have investigated a possible association between the APOE*4 allele and the presence of psychotic symptoms in AD (AD+P)[4], but these study findings have been inconsistent: 9 studies reported a significant association, whereas 17 studies found no relationship or were otherwise inconclusive [1,4,5]. In this study, we systematically assessed 790 subjects with probable AD at baseline to investigate whether a relationship exists between the APOE*4 allele and four neuropsychiatric symptom (BPS) domains: hallucinations, delusions, agitation, and aberrant motor behavior.

## Materials and methods

### Subjects

All data for this study were obtained using protocols that were approved by the institutional review board at the University of Pittsburgh. The study initially included 812 patients from the University of Pittsburgh Alzheimer Disease Research Center (ADRC) who met the National Institute of Neurological and Communicative Disorders and Stroke–Alzheimer’s Disease and Related Disorders Association (NINCDS-ADRDA) clinical criteria for probable AD during the years 1992–2007. All subjects followed University of Pittsburgh ADRC protocol by participating in standardized neurological, psychiatric, neuropsychological, and functional evaluations, including the Mini-Mental State Examination (MMSE) and the Clinical Dementia Rating (CDR) scale [6]. Demographic data collected during the initial visit included gender, education, age and duration of illness. The MMSE and CDR scores used in this study correspond to subject visits that occurred within six months of the initial neuropsychiatric evaluation visit. All procedures were approved by an institutional review board and written informed consent was obtained from the patient for publication of this report.

### Neuropsychiatric symptom assessments

Neuropsychiatric symptoms were evaluated using the 1992 and 1996 versions of the Consortium to Establish a Registry for Alzheimer’s Disease (CERAD) Behavioral Rating Scale for Dementia (BRSD). The BRSD collects information from informants on six domains that are designed to quantify the severity of neuropsychiatric abnormalities in demented subjects [7,8]. In this study, we used ratings from the first assessment that was conducted with informants after subjects were initially diagnosed with probable AD. We focused on three neuropsychiatric domains generated from the BRSD items: psychosis (12 items), agitation (3 items), and aberrant motor behavior (2 items). Given that previous studies have reported conflicting findings concerning the relationship between the *APOE* ε4 allele and psychosis [1,4], we subdivided the psychosis category into two domains: hallucinations (2 items) and delusions (10 items). Hallucinations were defined as sensory perceptions that were not observed by others and delusions were defined as fixed false beliefs. Agitation was defined as uncooperative, verbally abusive, and physically aggressive behavior. Aberrant motor behavior was defined as restless, confused, or nonpurposeful activity. Neuropsychiatric symptoms that only occurred during an episode of delirium were not rated as present.

### *APOE* genotyping

The *APOE* three-allelic polymorphism was screened by PCR-based assay as previously described [9]. Genomic DNA were amplified using a forward primer E1, 5’-GCGGACATGGAGGACGTG-3’ and a reverse primer E2, 5’-GGCCTGGTACACTGCCAG-3’. The 177-nucleotide amplified product was digested directly with the restriction enzyme HhaI. The digested DNA was separated on 8% nondenaturing polyacrylamide gel in 1 X TBE buffer, followed by staining with ethidium bromide solution. The *APOE* polymorphism was then typed by visualization under UV light.

### Statistical analysis

Four neuropsychiatric domains were included in the analyses: hallucinations, delusions, agitation, and aberrant motor behavior. These domains were modeled as dichotomous: subjects for whom informants reported one or more items in a domain were classified as positive for that domain, whereas subjects for whom informants reported no occurrences of any item in a domain were classified as negative for that domain. Descriptive statistics of demographics and the neuropsychiatric domains are presented as mean and standard deviation (SD) for continuous variables and as frequency and percentage of group totals for categorical variables.

Differences in continuous variables by categorical variables were assessed using analysis of variance (the two-sample *t*-test for two-category variables), and associations between categorical variables were assessed using the chi-square test. Generalized linear regression with log link and error modeled as Gaussian was used to estimate the relative risk (RR) and 95% confidence intervals (CIs) of the occurrence of at least one abnormal neuropsychiatric symptom in a given domain according to the number of APOE*4 alleles (treated as categorical, with “no alleles” as the reference category) [10,11]. The model covariates were gender, education, age at BRSD assessment, baseline MMSE score, duration of illness, and baseline CDR score. Age, education, duration of illness, and MMSE were modeled as restricted cubic splines with three degrees of freedom to account for potential nonlinear trends [11,12], and CDR was modeled as categorical (0.5, 1, 2, 3+). If a significant association between behavioral domain and the number of APOE*4 alleles was found, a post-hoc analysis, modeling the number of APOE*4 alleles as continuous was carried out to determine if a dose–response effect was present. Confidence intervals were obtained through the construction of likelihood profiles for each parameter [13].

Classification of a domain as positive or negative was not possible for cases where at least one item in that domain was missing and the remaining items were either coded as 0 (i.e., the behavior or symptom was not present) or were also missing. For these cases, the domain was imputed to the median response of all individuals with non-missing domain values. As a sensitivity analysis, a second set of models was run to impute the missing domain values to the alternate values. For example, the hallucination domain consists of two items: auditory and visual hallucinations. Most individuals recorded having no hallucinations (i.e., median=0, no events), and thus, if an individual recorded no auditory hallucinations and gave no information regarding visual hallucinations, then for the primary analysis the hallucinations domain for that individual was coded as negative but for the sensitivity analysis the individual was coded as having at least one hallucination event.

A secondary analysis was used to compare subjects who experienced both hallucinations and delusions to subjects who only experienced delusions. This analysis examined the frequency and percent of subjects who experienced delusional domain items, broken down by item, from the 1996 version of the BRSD [8]. For each item, subjects with a missing value for that item were excluded from the analysis. All statistical analyses were carried out using R 2.11.1 [12,15].

## Results

Of the 812 subjects with AD who were originally considered for this study, 21 (5%) had *APOE* genotype Ε2Ε4. Because of the protective effects that have been previously reported for the APOE*2 allele [15], individuals with the Ε2Ε4 genotype were excluded from this analysis, although including them in the analyses produced similar results to those presented below. An additional subject with no neuropsychiatric data available was also excluded, leaving 790 subjects for the analysis. Table 1 shows that 348 subjects (44%) had no APOE*4 allele, 368 (47%) had one Ε4 allele, and 74 (9%) had two Ε4 alleles. The sample was 33% male and had a mean education of 12 years, a mean illness duration of 4 years, a mean MMSE score of 17.6, and a mean CDR score of 1.29. The mean age at assessment decreased in relation to the number of APOE*4 alleles subjects possessed, from 78.1 years (SD 5.9) for those with no APOE*4 alleles to 72.8 years (SD 5.4) for those with two APOE*4 alleles, but otherwise there was no evidence of an association between the demographic variables and the number of APOE*4 alleles.

**Table 1:** Demographics and clinical characteristics of Alzheimer’s disease study sample

	No APOE*4 alleles (n=348)	One APOE*4 allele (n=368)	Two APOE*4 alleles (n=74)	P*
Male, frequency (%)	111 (32%)	116 (32%)	32 (43%)	.131
Age at assessment, mean [SD]	78.1 [5.9]	76.2 [5.8]	72.8 [5.4]	<.001
Duration of illness (years)	3.7 [2.5]	4.2 [2.9]	3.9 [2.8]	.077
Education (years)	12.5 [3.1]	12.8 [3.0]	13.1 [2.7]	.208
Baseline MMSE	17.5 [5.8]	17.6 [5.2]	17.5 [5.8]	.960
Baseline CDR	1.31 [0.69]	1.29 [0.63]	1.24 [0.67]	.623

*Chi-square tests for differences in gender by genotype, and ANOVA for differences in means across genotype for all other demographic measures.

Abbreviations: *APOE*, apolipoprotein E; MMSE, Mini-Mental State Examination; CDR, Clinical Dementia Rating scale; ANOVA, analysis of variance.

Table 2 shows the frequency of reported neuropsychiatric symptoms in the four domains by number of APOE*4 alleles and the RR of a neuropsychiatric symptoms in a given domain by one or two APOE*4 alleles versus no APOE*4 alleles. Of the 790 subjects, 110 (14%) had missing values for at least one item in the BRSD. The occurrence of hallucinations was imputed for 6 (0.8%) subjects, the occurrence of delusions was imputed for 101 (13%) subjects, and the occurrence of aberrant motor behavior was imputed for 9 (1%) subjects; there were no missing agitation values, so no imputation was necessary for this domain. Across all 790 subjects, 99 (13%) experienced at least one occurrence of hallucinations, 540 (68%) experienced an occurrence of delusions, 327 (41%) experienced an occurrence of agitation, and 426 (54%) experienced an occurrence of aberrant motor behavior. In comparing subjects with different numbers of APOΕ*4 alleles, the occurrence of hallucinations tended to decrease as the number of APOΕ*4 alleles increased; we found that 53 (15%) subjects with no APOΕ*4 alleles experienced hallucinations, whereas only 4 (5%) subjects with two APOΕ*4 alleles experienced hallucinations. These results are illustrated by the decreasing RRs of hallucinations for one APOΕ*4 allele (0.71, 95% CI [0.50, 0.97]) and two APOΕ*4 alleles (0.32, 95% CI [0.00, 0.74]) versus no APOΕ*4 alleles after adjusting for age, gender, education, duration of illness, MMSE score, and CDR score at assessment. A post-hoc analysis modeling the number of APOE*4 alleles as continuous yielded a test for trend p-value of 0.0021 and an estimated relative decrease in risk of hallucinations of 34% for each increase in one APOE*4 allele, that is, a relative risk of 0.66, 95%CI (0.49, 0.86). There was no evidence of associations between the number of APOΕ*4 alleles and the occurrence of neuropsychiatric symptoms in the other three domains. Sensitivity analyses that imputed domains with missing items to the alternate values yielded similar results.

**Table 2:** Frequency of AD subjects with at least one neuropsychiatric symptom in a given domain, and relative risk for the symptom in that domain by one and two APOΕ *4 alleles versus no APOE*4 allele

**Domain**	No APOΕ *4 allele (n=348)	One APOΕ *4 allele (n=368)	Two APOΕ *4 alleles (n=74)	P*
Hallucinations (2 items)				
Frequency (%)	53 (15%)	42 (11%)**	4 (5%)	
RR 95% CI	1.00	0.71 (0.50, 0.97)	0.32 (0.00, 0.74)	0.0066
Delusions (10 items)				
Frequency (%)	241 (69%)	259 (70%)	40 (54%)	
RR 95% CI	1.00	1.05 (0.96, 1.15)	0.87 (0.72, 1.07)	0.160
Agitation (3 items)				
Frequency (%)	150 (43%)	147 (40%)	30 (41%)	
RR 95% CI	1.00	0.93 (0.79, 1.11)	1.04 (0.77, 1.35)	0.628
Aberrant motor behavior (2 items)				
Frequency (%)	196 (56%)	195 (53%)	35 (47%)	
RR 95% CI	1.00	0.94 (0.83, 1.07)	0.86 (0.67, 1.08)	0.382

*Generalized linear models of domain on the number of *APOΕ* *4 alleles (treated as categorical) with log link, and error modeled as Gaussian and with the following covariates: age, duration of illness, MMSE, CDR, gender, and education.

**2 subjects with 1 *APOE* 4 allele were missing all hallucination domain data.

Abbreviations: *APOE*, apolipoprotein E; RR, relative risk; CI, confidence interval; MMSE, Mini-Mental State Examination; CDR, Clinical Dementia Rating scale; BRSD, Behavioral Rating Scale for Dementia.

**Figure 1:**
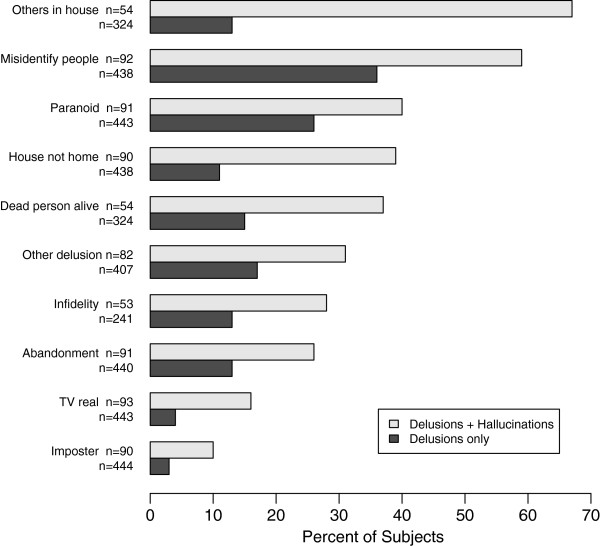
**Frequency of Delusion Type by Group.** Legend: Frequency of specific delusion items by group (delusions and hallucinations vs. delusions only). This figure demonstrates that the delusions and hallucinations group had statistically significant higher frequencies for every kind of delusions compared to the group that experienced delusions only.

Of the 99 subjects who experienced hallucinations, 93 (94%) experienced at least one delusion as well, and of the 538 subjects who experienced delusions and had non-missing values for hallucinations, 445 (83%) experienced no hallucinations. The RR of delusions without hallucinations was 1.14 (95% CI [1.00, 1.29]) for subjects with one APOE*4 allele and 1.00 (95% CI [0.77, 1.29]) for subjects with two APOE*4 alleles versus subjects with no APOE*4 alleles, in contrast to the RR of hallucinations *and* delusions of 0.71, (95% CI [0.52, 0.97]) and 0.34 (95% CI [0.11, 1.05]) for one and two APOE*4 alleles respectively vs. no alleles. We conducted a secondary analysis comparing the 445 subjects with delusions and no hallucinations (D) to the 93 subjects with delusions and hallucinations (D+H). Our findings, summarized in Table 3, show that the D+H group had a significantly longer duration of illness, lower MMSE scores, and higher CDR scores compared to the D group. On the other hand, there were no significant differences in gender, age at assessment, and education between the two groups. Although there was no significant difference in APOΕ*4 allele frequency between the two groups, the D+H group tended to have lower APOΕ*4 frequencies than the D group. As Figure 1 illustrates, the D+H group had significantly higher frequencies for each kind of delusion item than the D group. All comparisons were statistically significant (paranoid and imposter, *P*<.05, all others, *P*<.01).

**Table 3:** Demographic and clinical characteristics of AD subjects with delusions only compared to subjects with delusions and hallucinations

	Delusions without hallucinations (D) (n=445)**	Delusions and hallucinations (D+H) (n=93)**	*P *value*
Male, frequency (%)	129 (29%)	19 (20%)	.120
*APOE* ε4 allele frequency			.116
0 *APOE* ε4 alleles	191 (43%)	50 (54%)	
1 *APOE* ε4 allele	218 (49%)	39 (42%)	
2 *APOE* ε4 alleles	36 (8%)	4 (4%)	
Age at assessment, mean (SD)	77.4 (5.8)	77.7 (6.9)	.732
Duration of illness (years)	4.1 (2.6)	5.0 (3.2)	.010
Education (years)	12.3 (2.9)	11.8 (2.5)	.091
Baseline MMSE	17.1 (5.6)	15.4 (6.0)	.010
Baseline CDR	1.35 (0.65)	1.75 (0.76)	<.001

*Chi-square test for gender and allele frequency; *t*-test for all others.

** 2 subjects missing all hallucination domain data are omitted from this analysis.

Abbreviations: *APOE*, apolipoprotein E; SD, standard deviation; MMSE, Mini-Mental State Examination; CDR, Clinical Dementia Rating scale.

## Discussion

In this study of AD, we found a significant dose-dependent relationship between the number of APOE*4 alleles and hallucinations but no association between APOE*4 and aberrant motor behavior or agitation. We also found important differences between AD patients with delusions alone and AD patients who experienced a co-occurrence of hallucinations and delusions at the time of baseline assessment. In previous studies, hallucinations and delusions were usually combined and categorized under the broad category of psychosis [16], and the largest of these studies have shown no association between APOE*4 and a broad category of psychosis [5,17]. In a recent genome-wide association study (GWAS), the odds ratio (OR) was 1.09 for the rs2927438 SNP in the APOE*4 locus that showed the strongest association between AD with and without psychosis, but this association did not reach statistical significance (p=.306) [5] However, the current study illustrates that these modest genetic effects might be obscured when the psychosis phenotype cannot be further subdivided (e.g., D+H or D alone). The findings of the present study suggest that psychotic AD subjects who experience hallucinations and delusions may comprise a genetic category that is distinct from those subjects who experience delusions exclusively. These results are partially supported by several studies that have previously looked at the possibility of subphenotypes within the broader category of AD+P [16,18]. In order to analyze this possible distinction, we classified subjects into two non-overlapping groups in this study so that each individual patient belonged to only one group: given that nearly all of the subjects who experienced hallucinations also experienced delusions, one of those groups included subjects who experienced both delusions and hallucinations (D+H) and the other included subjects who experienced D alone. Under the aforementioned two-group classification model, we found that AD patients with D+H were more severely affected than the AD patients with D alone (i.e., the patients with D+H had a longer duration of illness, lower baseline MMSE scores, and higher CDR scores than patients with D alone). Although it is possible that the D+H subjects were simply farther along in their stage of disease than the D alone subjects, both groups were in the same stage of dementia (moderate) and the differences in MMSE and CDR between the two groups were not clinically meaningful.

Interestingly, the D+H subjects also experienced significantly higher frequencies of all types of delusions compared to subjects with D alone. Of note, delusions of misidentification, which are typically associated with dementia with Lewy bodies, were reported in 63% of subjects with D+H. Unfortunately, we do not have sufficient neuropathological information to determine whether these subjects have Lewy body related and AD pathologies.

After controlling for potential confounders, we consistently found a protective effect of the APOE*4 allele against D+H. We have previously proposed that there are potentially two plausible genetic models of AD with D+H [1]: first, a heterogeneity model in which alleles predispose or are protective against the development of AD pathology and subsequent psychosis and, second, a disease-modifier model in which alleles increase the risk of psychosis but only in the presence of AD. A recent GWAS in AD + P found no support for either model, as there was no genome-wide significant finding for AD + P when compared to controls (testing model 1) or between AD with and without psychosis (testing model 2) [5]. However, even though that GWAS included the largest available cohort of AD with (n=1299) and without psychosis (n=735), the power to observe alleles of small effect was limited. Another explanation for the lack of significant GWAS findings may be that the susceptibility to develop psychosis is due to nongenetic familial factors.

One limitation of this study is that we used proxy reporting instead of direct observation to assign the presence or absence of neuropsychiatric symptoms in each of the four domains we looked at. Previous studies have shown discrepancies in the accuracy of proxy reporting for assessing behavioral symptoms of dementia patients when compared with the use of direct observation [19]. Although direct observation would have been the preferred method, the size and nature (comprised entirely of outpatient subjects) of our observed population made this impractical. Another limitation is due to missing values that required imputing the presence or absence of neuropsychiatric symptoms for a specific domain. However, our main finding involved hallucinations, which required imputation for less than 1% of the subjects. Furthermore, sensitivity analyses in which imputed values were set to the alternative value did not change our findings. Another limitation is that the variability observed in the role of ΑPOE*4 with regard to D+H and AD can at least be partially accounted for by differences in study design, the size of the cohort examined, the cognitive and neuropsychiatric tests used, and the tools and methods by which data were analyzed. In this study, for example, we noted a large degree of variation between our subjects in the number of follow-up assessments and in the time between follow-up assessments, and therefore we opted to analyze baseline assessments rather than longitudinal assessments. It is possible, therefore, that our findings concerning ΑPOE*4 and psychosis may have differed in a longitudinal data set. As mentioned previously, we attempted to account for these kinds of differences by including CDR, MMSE, and other indicators of disease stage as covariates in our analysis model. Another limitation of our study design, as in any case–control genetic association study, is the possibility of spurious associations, and thus these findings should be replicated in other large samples and in longitudinal studies.

An important strength of our study is our rigorous clinical assessment of behavioral symptoms. Together with the size of our sample—one of the largest of its kind—and our use of AD research subjects who were recruited from a single geographical site, the comprehensiveness of the assessments enabled us to differentiate the subtypes of psychosis. However, the underlying mechanism by which *APOE* affects the occurrence of hallucinations and delusions has yet to be elucidated.

## Conclusion

In the present study we found that hallucinations were significantly more likely to occur in subjects with no *APOΕ*4 alleles than in subjects with two Ε4 alleles. Furthermore, our findings suggest that in AD the Ε4 allele is differentially associated with D+H but not delusions alone. This is consistent with our proposed hypothesis that distinct psychotic subphenotypes may be associated with the *APOE* allele. Future studies should take into account that the psychotic phenotype in AD patients may not be homogenous, but consist rather of subphenotypes of hallucinations and delusions that in turn may have different genetic associations.

## Abbreviations

AD: Alzheimer’s Disease; LOAD: Late-onset Alzheimer’s Disease; *APOE*: Apolipoprotein E; AD+P: Alzheimer’s Disease with psychosis; D+H: Subjects that experienced both delusions and hallucinations; D: Subjects that experienced delusions exclusively; BRSD: Behavioral Rating Scale for Dementia; MMSE: the Mini-Mental State Examination; CDR: Clinical Dementia Rating scale.

## Competing interests

We declare no actual or potential competing interests.

## Authors’ contributions

OL and RS obtained funding and evaluated the subjects at the University of Pittsburgh's Alzheimer’s Disease Research Center that contributed data to this study. JS and EL drafted the initial manuscript. DC drafted the final manuscript and reviewed the literature for this manuscript. RS, DT, and SM participated in the design of the study. DT, MS and EW also contributed to subject tracking, scheduling, and collection of data, including conducting CERAD ratings. JS and SM performed the statistical analysis. IK carried out the molecular genetic studies. All authors read and approved the final manuscript.
